# Brief communication: targeted serum proteomics in postpartum South African women living with and without HIV, correlations with anthropometry and adiposity

**DOI:** 10.1186/s12981-025-00782-0

**Published:** 2025-08-04

**Authors:** Hlengiwe P. Madlala, Junyu Chen, Jennifer Jao, Landon Myer, Amy E. Mendham, Carmen Pheiffer, Liam Bell, Lara R. Dugas, Julia H. Goedecke, Yan V. Sun, Angela M. Bengtson

**Affiliations:** 1 Division of Epidemiology and Biostatistics, School of Public Health, University of Cape Town, Falmouth Building, Anzio Road, Observatory, Cape Town, 7925 South Africa; 2 Department of Epidemiology, Rollins School of Public Health, Emory University, Atlanta, GA USA; 3 Division of Infectious Diseases, Department of Pediatrics & Department of Medicine, Northwestern University Feinberg School of Medicine, Chicago, IL USA; 4 Riverland Academy of Clinical Excellence (RACE), Riverland Mallee Coorong Local Health Network, South Australia Health, Berri, SA Australia; 5 Division of Physiological Sciences, Department of Human Biology, Faculty of Health Sciences, Health through Physical Activity, Lifestyle and Sport Research Centre (HPALS), FIMS International Collaborating Centre of Sports Medicine, University of Cape Town, Cape Town, South Africa; 6 Non-Communicable Disease Research Unit, South African Medical Research Council, Cape Town, South Africa; 7 Department of Obstetrics and Gynecology, Faculty of Health Sciences, University of Pretoria, Johannesburg, South Africa; 8 D-CYPHR Regional Hub, Centre for Proteomic and Genomic Research, University of Cape Town, Cape Town, South Africa; 9 Public Health Sciences, Parkinson School of Health Sciences & Public Health, Loyola University Chicago, Maywood, IL USA; 10 Biomedical Research and Innovation Platform, South African Medical Research Council, Cape Town, South Africa; 11 Department of Global Health, Rollins School of Public Health, Emory University, Atlanta, GA USA; 12 Division of Cardiology, Department of Medicine, School of Medicine, Emory University, Atlanta, GA USA

**Keywords:** Adiposity, HIV, Postpartum, Proteomics, Women, South africa

## Abstract

Postpartum adiposity is associated with long-term risk of cardiovascular disease (CVD), which may be influenced by HIV infection. We investigated cross-sectional correlations between postpartum adiposity measures, and circulating proteins linked to CVD in women living with and without HIV. Irrespective of HIV status, adiposity was positively correlated with fatty acid binding protein 4, leptin and galectin 9; and negatively correlated with insulin-like growth factor binding protein 2 and V-set immunoglobulin domain containing 2. The link between adiposity and CVD-linked circulating proteins underscores the role of protein markers in understanding the cardiometabolic health of postpartum women living with and without HIV. *Clinical trial number*: Not applicable.

**Supplementary Information:**

The online version contains supplementary material available at 10.1186/s12981-025-00782-0.

## Introduction

Postpartum adiposity is a risk factor for future cardiovascular disease (CVD) [1]. Women who gained 6% of their pre-pregnancy weight 5 years after delivery exhibited a worsening CVD risk profile compared to those who lost weight [1]. In South Africa, women with HIV (WWH) and without HIV tend to gain rather than lose weight during the first 12 months postpartum [2] which may increase their risk of CVD over time.

Circulating proteins may reflect early disruptions in metabolic pathways, are known to be influenced by adiposity and HIV infection, and can serve as potential biomarkers of CVD [3, 4]. Specifically, high levels of circulating proteins associated with worse CVD profile have been shown in persons living with HIV, including those on life-long antiretroviral therapy (ART), compared to HIV-negative counterparts [3, 5]. However, the relationship between adiposity and CVD-related proteomic biomarkers remains underexplored in sub-Saharan African women, particularly in the context of HIV infection and the postpartum period.

To help address this knowledge gap, we investigated cross-sectional associations between postpartum anthropometry, adiposity, and circulating CVD-related proteins, both overall and modification by HIV status and ART regimen.

## Methods

### Study participants

A total of 84 women (58 WWH; 26 without HIV) aged ≥ 18 years from a low-resourced Gugulethu primary healthcare clinic in Cape Town were enrolled from a larger ‘CArdioMetabolic complications in Pregnancy’ (CAMP) study [6]. WWH were using efavirenz (EFV)- or dolutegravir (DTG)-based ART. For this sub-study, we conducted a cross-sectional analysis at 6–24 months postpartum. An overview of participant numbers is shown in Figure S1. Study procedures were approved by the Faculty of Health Sciences Human Research Ethics Committee of the University of Cape Town (653/2020 and 505/2020).

### Anthropometry, adiposity and proteomics

Anthropometry measures included weight, body mass index (BMI), and waist and hip circumferences. Adiposity defined as total body composition (fat mass [FM], fat-free mass, body fat%) and regional fat distribution (FM % of android and gynoid, as well as abdominal subcutaneous [SAT] and visceral [VAT] adipose tissue) was measured using dual-energy X-ray absorptiometry (DXA) (Hologic Discovery-W, Bedford, USA) [7]. A targeted proteomic analysis of circulating proteins in fasted serum samples was performed at the D-CYPHR (https://www.d-cyphr.org.za) using CVD-II and CVD-III Olink panels of 96 proteins (https://olink.com/products/olink-target-96). These panels include targeted cardiovascular and cardiometabolic proteins involved in inflammation, immune response, chemotaxis, angiogenesis and the mitogen-activated protein kinase cascade. Of the 192 proteins tested, *n* = 26 had expression lower than limit of detection and therefore were excluded from the analysis.

### Covariates

Maternal age and self-reported pre-pregnancy weight were assessed at 24–28 weeks gestation and breastfeeding status was evaluated at 6–24 months postpartum. Measured pre-pregnancy weight is not typically available in this setting. Self-reported pre-pregnancy weight has been shown to be similar to measured weight [8], and was used to calculate pre-pregnancy BMI.

### Statistical analysis

Linear regression models estimated associations between adiposity measures and protein levels. Protein outcomes were expressed in normalized protein expression (NPX) units. All models were adjusted for HIV status, age, pre-pregnancy BMI, breastfeeding status and months postpartum as a priori confounders based on theoretical/biological reasoning. Variance inflation factor analysis excluded problematic multicollinearity among these covariates. Benjamin-Hochberg correction for multiple testing was used and adjusted p-values < 0.05 are reported. For identified correlations, modification by HIV status and ART regimen (EFV vs. DTG) was evaluated using interaction terms. Data were analysed using STATA version 15.0 (Stata Corporation, College Station, TX, USA) and R Studio (R Foundation, Vienna, Austria).

## Results

The median age was 31 years (SD = 5.94), postpartum BMI was 31 kg/m^2^ (SD = 7.25), and time since delivery was 12 months (SD = 5.24) (Table S1).

Overall, total and central SAT (weight, waist circumferance, FM and abdominal SAT) were positively correlated with fatty acid binding protein 4 (FABP4) (Table 1). Weight was also correlated with galectin 9 (Gal.9). Additionally, total and peripheral adiposity (BMI, hip circumference, FM, total body fat % and abdominal SAT) were positively correlated with leptin. Visceral adiposity (VAT and VAT/SAT ratio) were negatively correlated with V-set and immunoglobulin domain containing 2 (VSIG2) and insulin-like growth factor binding protein 2 (IGFBP.2). Non-significant correlations with all other proteins are presented in Table S2.

**Fig. 1:**
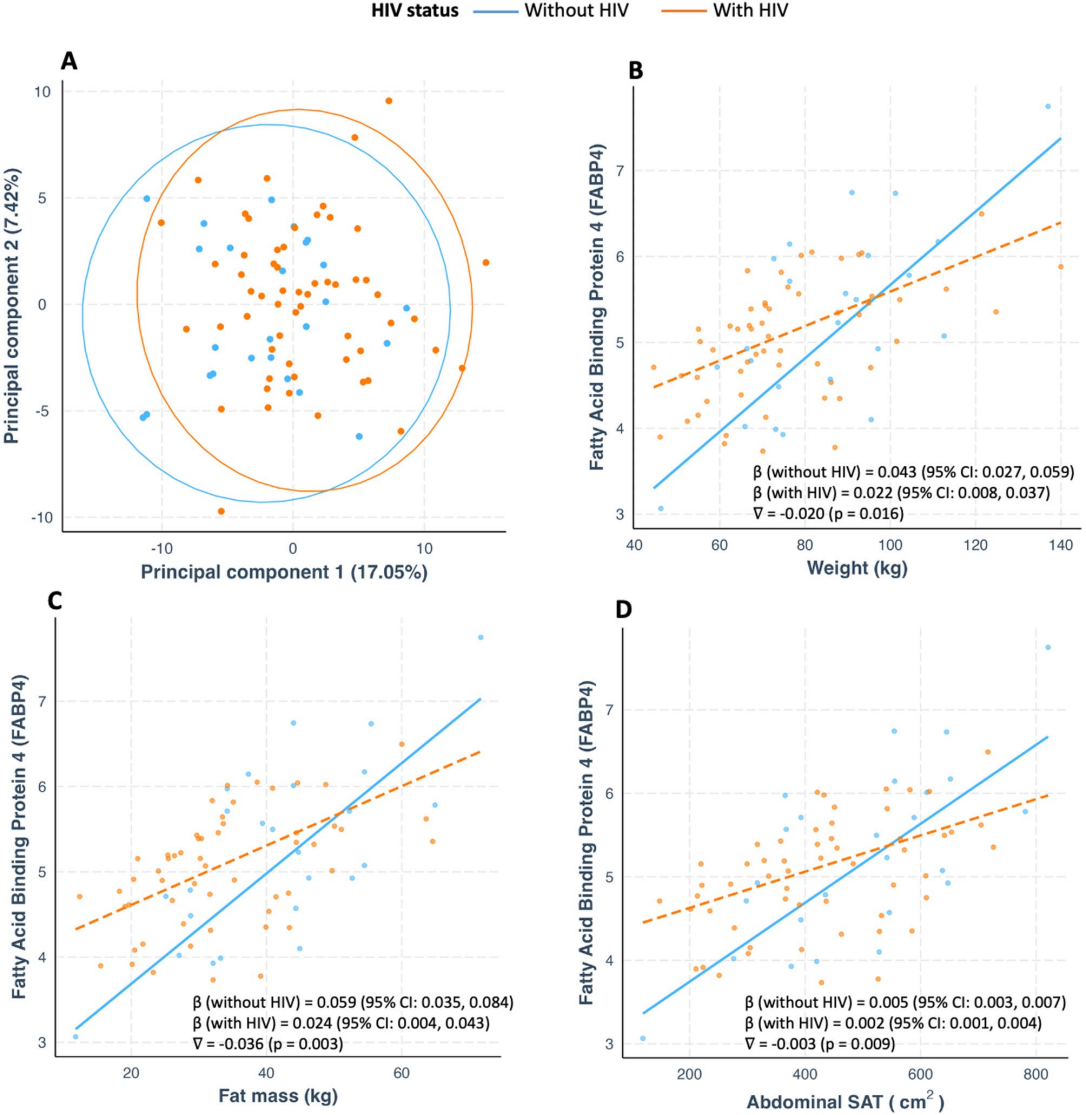
Principal component analysis by HIV status (**A**), HIV modification of the correlation between weight (**B**), fat mass (**C**) and abdominal SAT (**D**) and circulating fatty acid binding protein 4; nabla (∇) represents an interaction between each adiposity parameter and HIV status. *SAT* subcutaneous adipose tissue

Profiles of 166 proteins assessed did not differ by HIV status (Fig. 1A). All identified correlations were not modified by HIV except for FABP4 which showed a weaker correlation with weight, FM and abdominal SAT in WWH compared to women without HIV (Fig. 1B-D). ART regimen did modify any of the observed associations (Table S3).

**Table 1:** Statistically significant correlations of postpartum anthropometry and adiposity with CVD-related circulating proteins in the overall sample

Model predictor	Model outcome (Protein)	Mean difference (95% CI)	Adjusted *p*-value (fdr-q)
Anthropometry			
Weight (kg)	FABP4	0.031 (0.018, 0.044)	< 0.01
	LEP	0.027 (0.013, 0.041)	< 0.01
	Gal.9	0.011 (0.005, 0.017)	< 0.01
BMI (kg/m^2^)	LEP	0.108 (0.060, 0.155)	< 0.01
Waist circumference (cm)	FABP4	0.031 (0.015, 0.047)	< 0.01
Hip circumference (cm)	LEP	0.043 (0.028, 0.059)	< 0.01
DXA-derived adiposity			
Fat mass (kg)	FABP4	0.035 (0.016, 0.054)	< 0.01
	LEP	0.038 (0.019, 0.057)	< 0.01
Body fat (%)	LEP	0.086 (0.055, 0.117)	< 0.01
Abdominal VAT (cm^2^)	VSIG2	– 0.008 (– 0.013,– 0.004)	< 0.01
	IGFBP.2	– 0.007 (– 0.011,– 0.003)	< 0.01
Abdominal SAT (cm^2^)	FABP4	0.003 (0.001, 0.004)	< 0.01
	LEP	0.003 (0.002, 0.005)	< 0.01
VAT-SAT ratio	VSIG2	– 5.707 (– 8.610,– 2.804)	< 0.01
	IGFBP.2	– 4.671 (– 7.204,– 2.138)	< 0.01

Adjusted for age, pre-pregnancy BMI, HIV status, breastfeeding and postpartum time

*BMI* body mass index, *CVD* cardiovascular disease, *CI* confidence interval, *DXA* dual-energy X-ray absorptiometry, *FABP4* fatty acid binding protein 4, *fdr-q* false discovery rate q-value, *Gal.9* galectin 9, *IGFBP.2* insulin-like Growth Factor Binding Protein 2, *LEP* leptin, *SAT* subcutaneous adipose tissue, *VSIG2* V-set and immunoglobulin domain containing 2, *WWH* women with HIV

## Discussion

These data demonstrate for the first time that multiple anthropometric and adiposity measures were positively correlated with FABP4, leptin and gal.9, and negatively correlated with VSIG2 and IGFBP.2 in postpartum WWH and without HIV living in a low-resourced setting in South Africa. These findings suggest that measures of postpartum adiposity correlate with circulating protein biomarkers linked to CVD, with few differences by HIV status, underscoring the need for further investigation into how HIV may influence CVD risk.

FABP4 is a lipid chaperone primarily expressed in adipocytes and macrophages [9]. Our findings align with previous research in the general population reporting higher FABP4 levels in women with obesity [9], and a reversal of this effect in patients who underwent a gastric bypass [10]. Causal associations between FABP4 and CVD events were demonstrated in large Swedish population-based prospective cohorts of 50–70 year olds [4]. Further, FABP4 levels were correlated with CVD risk in persons living with HIV [5]. In our study, WWH had weaker correlations between FABP4 and adiposity compared to women without HIV. Despite large differences in postpartum obesity by HIV status, adjusting for pre-pregnancy BMI did not explain this difference. One potential explanation is that WWH exhibited lower baseline levels of FABP4, which may have blunted adiposity-associated increases in FABP4 observed in those without HIV. Nonetheless, considering high rates of postpartum obesity in WWH and without HIV in this setting [2, 7], positive correlation between FABP4 and adiposity measures observed in this study may increase the risk of CVD.

Leptin is a pro-inflammatory adipokine produced by the adipose tissue, it’s primary role is the regulation of satiety/appetite and energy homeostasis [11]. In the context of obesity, leptin becomes dysregulated due to diminished response of body cells to leptin stimulus resulting in leptin resistance and associated overeating [11]. Consequently leptin is elevated in people with obesity and linked to CVD risk [12]. In the context of HIV, leptin has mainly been studied in relation to its potential contribution to metabolic disturbances in lipohypertrophic patients on older-generation thymidine analogue ART [3, 13, 14]. In the general population, conflicting findings were reported between leptin and central fat accumulation in those with and without HIV [3, 13, 14]. Our study in postpartum women using newer-generation ART showed that multiple measures of adiposity correlated positively with leptin, as also seen in those without HIV.

Galectin 9, a β-galactoside-binding lectin that regulates immune response, correlated positively with postpartum weight. Another study conducted in postpartum women found associations between Gal.9 and adiposity [15]. Although HIV infection did not alter this correlation in our study, Gal-9 plays a complex role in HIV by promoting reservoir formation, chronic inflammation, and viral reactivation [16]. Additionally, causal associations between Gal.9 and CVD events were demonstrated in middle-aged Swedish population [4]. These results highlight the need for monitoring adiposity in the postpartum period to identify women at high risk of CVD risk.

Circulating IGFBP.2 inhibits adipocyte differentiation and visceral fat, and is consistently lower in individuals with obesity across diverse cohorts [17]. Notably, we found an inverse relationship between visceral adiposity and IGFBP.2 in both WWH and without HIV. Other authors, however, reported that HIV-related inflammation suppresses IGFBP.2 thereby contributing to CVD development [18]. VSIG2 was also inversely correlated with visceral adiposity. This protein belongs to the immunoglobulin domain-containing family and is involved in lipid metabolism though it’s precise role remains largely unknown [19]. Although VSIG2 is expressed in visceral adipose tissue, no studies have evaluated its influence on adiposity measures.

Our study is not without limitations. There was a variation in the timing of the postpartum visit attendance (mostly due to COVID-19-related restrictions) which may have led to increased variability in adiposity measures, and potentially protein levels assessed. However, to account for this, we included postpartum time since delivery in all regression models. Also, we conducted a cross-sectional analysis and therefore cannot be certain about the direction of the associations reported; and no CVD endpoints were assessed. We did not have data on other important risk factors for CVD such as diet, smoking and physical activity. However, the use of high-throughput omics technique, robust measures of adiposity and inclusion of WWH is a significant strength.

In conclusion, among postpartum South African WWH and without HIV, measures of anthropometry and adiposity significantly correlated with circulating FABP4, leptin and galectin 9 previously linked to incident CVD events in other populations. These findings suggest that circulating proteins might have a potential to serve as biomarkers for CVD risk in young postpartum women years before the clinical development of CVD.

## Supplementary Information

Below is the link to the electronic supplementary material.

Supplementary Material 1

Supplementary Material 2

Supplementary Material 3

Supplementary Material 4

## Data Availability

All data generated or analysed during this study are included in this published article (and its supplementary information files). In addition, the datasets used and/or analysed during the current study are available from the corresponding author on reasonable request.

## Abbreviations

ART: Antiretroviral therapy
BMI: Body mass index
CVD: Cardiovascular disease
DTG: Dolutegravir
DXA: Dual-energy X-ray absorptiometry
EFV: Efavirenz
FABP4: Fatty acid binding protein 4
FM: Fat mass
Gal.9: Galectin
HIV: Human immunodeficiency virus
IQR: Interquartile range
IGFBP.2: Insulin-like growth factor binding protein 2
SAT: Abdominal subcutaneous adipose tissue
VAT: Visceral subcutaneous adipose tissue
VSIG2: V-set and immunoglobulin domain containing 2
WWH: Women with HIV
