# Supplementary figures and images for: Brief communication: targeted serum proteomics in postpartum South African women living with and without HIV, correlations with anthropometry and adiposity
Source: AIDS Res Ther. 2025 Aug 4;22:76. doi: 10.1186/s12981-025-00782-0 (PMC12320358; doi:10.1186/s12981-025-00782-0)

**
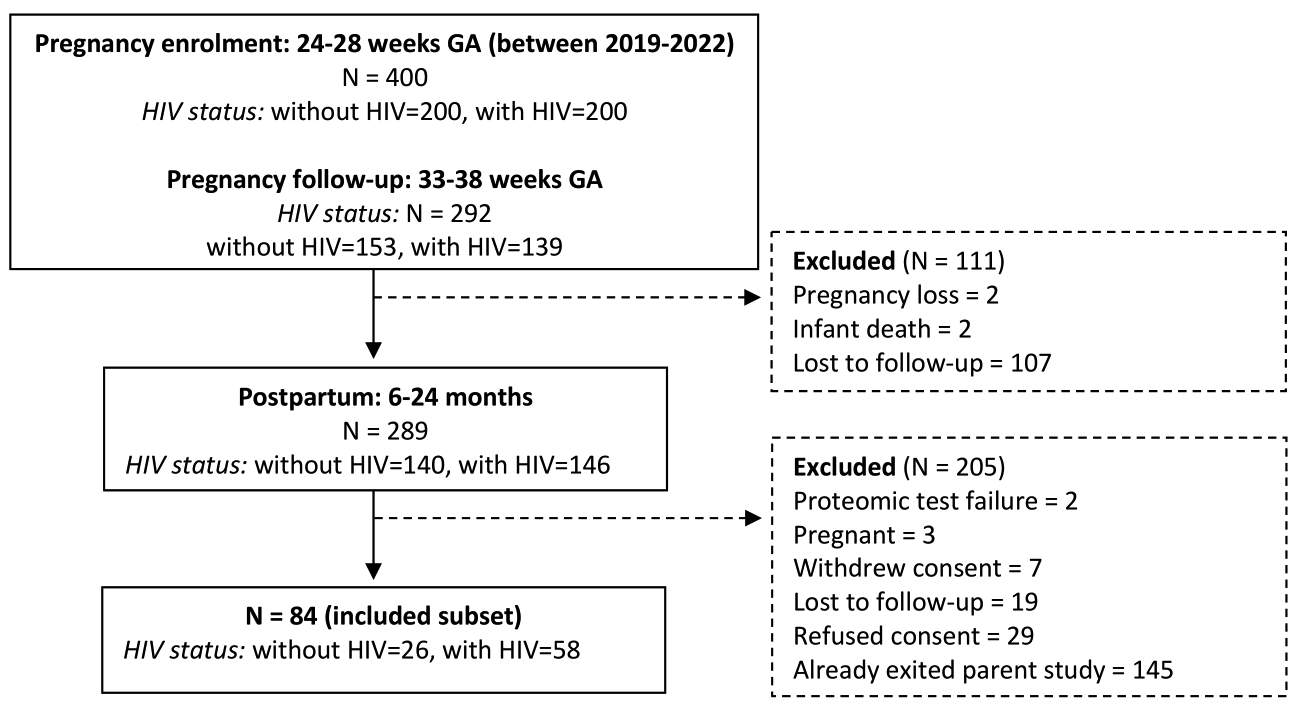
**

**Figure S1.** Participants included in this proteomics study. Abbrev: GA - gestational age

Supplement: Supplementary file 1 — Supplementary Material 1 [file 12981_2025_782_MOESM1_ESM.docx]
