# Supplementary material for: Brief communication: targeted serum proteomics in postpartum South African women living with and without HIV, correlations with anthropometry and adiposity
Source: AIDS Res Ther. 2025 Aug 4;22:76. doi: 10.1186/s12981-025-00782-0 (PMC12320358; doi:10.1186/s12981-025-00782-0)
Supplement: Supplementary file 2 — Supplementary Material 2 [file 12981_2025_782_MOESM2_ESM.docx]

Table S1. Participant characteristics and adiposity measures, overall and by HIV status

|  |  | HIV status | |
| --- | --- | --- | --- |
|  | Overall  (n = 84) | Without HIV  (n = 26) | WWH  (n = 58) |
| **Demographics** | N (%) | | |
| Still breastfeeding  Postpartum obesity (BMI ≥30 kg/m^2^)* | 31 (37)  38 (45) | 11 (42)  14 (37) | 20 (35)  24 (63) |
|  | Mean (±SD) | | |
| Age (years) | 30.50 (5.94) | 29.30 (6.22) | 31.1 (5.79) |
| Self-reported pre-pregnancy BMI (kg/m^2^) | 30.60 (6.44) | 32.10 (5.82) | 29.9 (6.64) |
| Postpartum time (months)* | 12.00 (5.24) | 15.00 (4.71) | 10.6 (4.90) |
| **HIV characteristics** | | | |
| ART regimen  EFV-based  DTG-based |  |  | 27 (47)  31 (53) |
| CD4 count (cells/µL)  ≤350  > 350  Mean (±SD) |  |  | 18 (36)  32 (64)  466 (218) |
| Viral Load (copies/mL)  Undetectable (<50)  Detectable (≥50)  Mean (±SD) |  |  | 13 (62)  8 (38)  16 162 (8591) |
| **Anthropometry** | | | |
| Weight (kg)* | 79.40 (20.00) | 84.80 (20.40) | 77.00 (19.50) |
| BMI (kg/m^2^)* | 30.80 (7.25) | 32.80 (7.17) | 29.90 (7.17) |
| Waist circumference (cm) | 97.60 (15.20) | 102.10 (17.50) | 95.60 (13.70) |
| Hip circumference (cm) | 111.40 (14.00) | 114.40 (14.00) | 110.10 (13.90) |
| Waist-hip ratio | 0.97 (0.85) | 0.89 (0.08) | 1.00 (1.02) |
| **DXA-derived body composition**  **and fat distribution** | | | |
| Fat mass (kg)* | 36.30 (13.60) | 40.80 (14.10) | 34.30 (13.0) |
| Fat-free mass (kg) | 40.30 (6.76) | 42.10 (6.54) | 39.50 (6.76) |
| Total body fat (%) | 46.20 (6.62) | 47.70 (7.48) | 45.60 (6.16) |
| Android FM (%) | 7.37 (1.36) | 7.38 (1.49) | 7.36 (1.31) |
| Gynoid FM (%) | 17.80 (2.22) | 17.50 (2.18) | 17.90 (2.25) |
| Abdominal VAT (cm^2^) | 126.00 (59.20) | 144.00 (66.50) | 118.00 (54.30) |
| Abdominal SAT (cm^2^)  VAT-SAT ratio | 453.00 (164.00)  0.27 (0.07) | 482 (177.00)  0.29 (0.08) | 440.00 (158.00)  0.26 (0.06) |
| * Indicates p<0.05. Missing data: CD4 count (n=8), viral load (n=37). Abbrev: BMI - body mass index, DTG - dolutegravir, DXA - dual-energy X-ray absorptiometry, EFV - efavirenz, FM - fat mass, SAT - subcutaneous adipose tissue, VAT- visceral adipose tissue, WWH - women with HIV | | | |
